# Supplementary material for: New Indicator of Arterial Stiffness START—Is There a Prognostic Value of Its Dynamics in Patients with Coronary Artery Disease?
Source: Biomedicines. 2024 Jul 23;12(8):1638. doi: 10.3390/biomedicines12081638 (PMC11351605; doi:10.3390/biomedicines12081638)
Supplement: Supplementary file 1 [file biomedicines-12-01638-s001.zip › biomedicines-3033790-supplementary.pdf]

Supplementary Table S1. Comparisons of survival between group with decreasing haSTART with increasing haSTART or no changes haSTART using Log Rank, Breslow, Tarone-Ware tests

Overall Comparisons			
	Chi-Square	df	Sig.
Log Rank (Mantel-Cox)	5.126	1	0.024
Breslow (Generalized Wilcoxon)	3.706	1	0.054
Tarone-Ware	4.065	1	0.044
Test of equality of survival distributions for the different levels of groups with improved index START and without improved index START			

Supplementary Table S2. Comparisons of event-free survival between group with decreasing haSTART with increasing haSTART or no changes haSTART using Log Rank, Breslow, Tarone-Ware tests

Overall Comparisons			
	Chi-Square	df	Sig.
Log Rank (Mantel-Cox)	2.257	1	0.133
Breslow (Generalized Wilcoxon)	1.175	1	0.278
Tarone-Ware	1.460	1	0.227
Test of equality of survival distributions for the different levels of groups with improved index START and without improved index START			
